# Estrogen and psychosis — a review and future directions

**DOI:** 10.1007/s00737-023-01409-x

**Published:** 2024-01-15

**Authors:** Eveline Mu, Caroline Gurvich, Jayashri Kulkarni

**Affiliations:** HER Centre Australia, Central Clinical School, Monash University, Melbourne, Victoria Australia

**Keywords:** Estrogen, Psychosis, Schizophrenia, Sex, Women, SERMs

## Abstract

The link between sex hormones and schizophrenia has been suspected for over a century; however, scientific evidence supporting the pharmacotherapeutic effects of exogenous estrogen has only started to emerge during the past three decades. Accumulating evidence from epidemiological and basic research suggests that estrogen has a protective effect in women vulnerable to schizophrenia. Such evidence has led multiple researchers to investigate the role of estrogen in schizophrenia and its use in treatment. This narrative review provides an overview of the effects of estrogen as well as summarizes the recent work regarding estrogen as a treatment for schizophrenia, particularly the use of new-generation selective estrogen receptor modulators.

## Introduction

Schizophrenia, a complex and debilitating condition, is associated with distinct sex differences. Decades of research have established a link between sex hormones, notably estrogen, and schizophrenia. Both animal and human studies have demonstrated estrogen’s profound impact on the central nervous system, specifically on the neurotransmitter systems implicated in the development of schizophrenia. The observations of sex differences in the age of onset of schizophrenia, as well as the exacerbation, relapse, or new onset of schizophrenia at times when estrogen levels drop (e.g., premenstrual or menopause), led to the hypothesis that estrogen may have a protective effect against schizophrenia, and clinical trials to date on the use of adjunctive estradiol support this notion. This narrative review aims to present an updated overview of the research in schizophrenia pertaining to sex differences, the role of estrogen, estrogen and hyperprolactinemia, and estrogen as a treatment. Additionally, future research and clinical directions are provided.

## Sex differences in schizophrenia

The onset and course of schizophrenia vary noticeably between men and women (Hafner 2003; Hafner and van der Heiden 1997; Gogos et al. 2015). Generally, schizophrenia affects men slightly more frequently than women, with a ratio of nearly 1.4:1 (Jongsma et al. 2019), and men experience an earlier onset of symptoms. The peak age of onset for men with schizophrenia is between 20 and 29 years, while for women, this peak occurs about 5 years later between ages 20 and 39 (Giordano et al. 2021). Additionally, women tend to exhibit a slightly lower incidence of psychosis and less social and clinical disability compared to men (Van Der Werf et al. 2014; Ochoa et al. 2012). However, this trend appears to shift in late years, with women showing higher incidence rates after the age of 40 (Van Der Werf et al. 2014; Riecher-Rössler et al. 1997), which may be related to decreased estrogen levels after menopause. Although women tend to attempt suicide more frequently, complete suicide rates are lower compared to men (Wang et al. 2020).

Earlier menarche — that is, earlier rise of estrogen levels — seems to be associated with a later onset of schizophrenia (Cohen et al. 1999; Kiliçaslan et al. 2014). Early work by Cohen et al. (1999) examined the relationship between pubertal age and psychosis onset in both men and women. The authors reported statistically significant negative correlations between puberty age and measures of schizophrenia onset (first odd behavior, first psychotic symptoms, and first psychiatric hospitalization) in women, but no significant associations were found in men (Cohen et al. 1999). Psychotic symptoms also often deteriorate premenstrually or perimenstrually (Handy et al. 2022; Reilly et al. 2020). During these phases of low estrogen, women experience an exacerbation or worsening of psychotic manifestations, presenting challenges in symptom management and overall mental well-being (Gogos et al. 2015; Reilly et al. 2020). Previous studies examining self-reported menstrual exacerbations reported prevalence ranging from 20 to 32.4% (Gleeson et al. 2016; Hsiao et al. 2004). Psychiatric admissions are also significantly greater during the perimenstrual phase of the cycle, with studies showing not only a significant excess of patient admissions during this phase but also an inverse correlation of estradiol concentrations in the blood with psychopathology (Reilly et al. 2020). When estradiol concentrations in the blood increased naturally during the cycle, there appeared to be an improvement in psychotic and total symptomatology, and vice versa.

Studies across the life cycle highlight women’s heightened vulnerability, compared to men, regarding either the onset of a first episode or relapse of existing illness during two significant hormonal shifts: the postpartum period and menopause, both marked by declining estrogen levels (Seeman 1986; Seeman 1996). After menopause, women with pre-existing chronic schizophrenia tend to experience a worsening of their condition, leading to an increased requirement for antipsychotic medication. Additionally, the decline in estrogen levels can diminish the production of enzymes responsible for metabolizing antipsychotic medications, consequently reducing their effectiveness. Beyond psychotic symptoms, estrogen loss can exacerbate various conditions, including sleep disturbances, irritability, depression, cognitive impairment, and sexual problems. While estrogen replacement, like estradiol, may offer relief during the perimenopausal and early postmenopausal phases in women with schizophrenia, its usage may entail certain risks. A recent study by Sommer et al. (2023) showed that the proportion of patients requiring hospitalization for psychosis is even among men and women until the age of 45. However, after 45, more women than men with psychosis need hospitalizations — coinciding with perimenopause.

Symptoms of schizophrenia also manifest differently in males and females. Typically, males diagnosed with schizophrenia tend to exhibit more pronounced negative symptoms such as anhedonia and avolition (Schultz et al. 1997; Li et al. 2016). They also often experience greater impairments in occupational and social functioning (Hanlon et al. 2017; Rietschel et al. 2017). On the other hand, females with schizophrenia tend to present with higher rates of affective symptoms (Mancuso et al. 2015), including comorbid depression and anxiety. Additionally, research has suggested that females may be more prone to engage in suicidal behaviors (Heila et al. 1997). In relation to sex differences in cognition in schizophrenia, females tend to have an advantage in verbal learning and memory and males tend to have an advantage in spatial tasks; however, similar sex differences are observed in healthy controls indicating sex-specific advantages for certain cognitive functions remain unaffected by schizophrenia (Leger and Neill 2016; Zhang et al. 2017; Menghini-Müller et al. 2020).

## The role of estrogen in schizophrenia

The aforementioned sex differences in schizophrenia relate to the hypothesis that estrogen, especially estradiol, possesses neuroprotective qualities against psychosis (Saldanha 2020; Sbisa et al. 2018; McGregor et al. 2017).

Schizophrenia’s pathogenesis is often viewed as involving a progressive neurodegenerative element (Iritani 2007). The brains of those with schizophrenia display a range of anatomical irregularities, encompassing reduced grey matter volume in frontal, temporal, limbic, striatal, and thalamic regions, as well as ventricular enlargement, and anomalies in the medial temporal lobe and prefrontal cortex (Kuo and Pogue-Geile 2019; Dabiri et al. 2022; Buckley 2005; Fornito et al. 2009; Shenton et al. 2001; Pomarol-Clotet et al. 2010). Estrogen possesses diverse neuroprotective attributes, which may be especially relevant in influencing the onset and progression of brain disorders in schizophrenia. In vivo and in vitro studies have shown that estrogenic compounds can protect brain cells from damage caused by excitotoxicity, oxidative stress, inflammation, and apoptosis (Arevalo et al. 2011; Arevalo et al. 2010; Bryant and Dorsa 2010; Yang et al. 2010; Vedder et al. 1999). Moreover, they have the capacity to enhance processes such as neurogenesis, angiogenesis, synaptic density, plasticity, connectivity, axonal sprouting, remyelination, and the expression of neurotrophic factors (Yang et al. 2010; Riecher-Rössler and Häfner 1993; Li et al. 2011; Liu et al. 2010; Diotel et al. 2013; Trenti et al. 2018; Mahmoud et al. 2016). It is thought that these neuroprotective mechanisms are primarily mediated by the action of neuronal estrogen receptor-α (Elzer et al. 2010). Other findings also propose that the neuroprotective qualities of estrogens may arise from their preservation and enhancement of neuronal mitochondrial function during injurious circumstances, as mitochondria are involved in regulating the survival and death of neurons (Simpkins et al. 2010; Klinge 2020) and may be impaired in the brains of individuals with schizophrenia (Rezin et al. 2009; Ni and Chung 2020; Cuperfain et al. 2018; Kulkarni et al. 2019).

Moreover, estrogens increase dopamine sensitivity of dopamine D2/D3 receptors in the ventral tegmental area (VTA) (Brand et al. 2021; Vandegrift et al. 2017) that reduce psychotic symptoms. Circulating estrogens also modulate other neurotransmitter systems relevant to schizophrenia, such as the serotonergic, glutamatergic, noradrenergic, and cholinergic systems (Gogos et al. 2015; Steeds et al. 2015; Hwang et al. 2020). Some authors have even suggested that estradiol in the brain should be regarded as a neurotransmitter itself (Balthazart and Ball 2006; Brann et al. 2022). Another potential rationale for the protective mechanism of estrogens could be attributed to the influence of heightened estrogen levels on regulating hippocampal plasticity (Adams et al. 2001; Sheppard et al. 2019; Finney et al. 2020). This modulation may lead to a decrease in the typical hippocampal dysconnectivity observed in psychosis (Bahner and Meyer-Lindenberg 2017). Damme et al. (2020) examined whether earlier age of menarche would result in more normative hippocampal connectivity in clinical high-risk youth. They found that later age at menarche in the clinical high-risk group related to increased hippocampal dysconnectivity to the occipital cortex — a region with a neurotrophic response to estrogen (Damme et al. 2020). However, more recent studies have failed to replicate a similar association between the age at menarche and age of illness onset (Barrau-Sastre et al. 2022; Fassler et al. 2019).

## Estrogen and hyperprolactinemia

Antipsychotic medications are frequently responsible for hyperprolactinemia (Gonzalez-Rodriguez et al. 2020), which is the result of the blockade of dopamine receptors on prolactin-releasing cells in the anterior pituitary gland (Rubin, and T, Prolactin and schizophrenia 1987). When these cells are freed from the inhibition of dopamine, more prolactin is secreted, and this can lead to reduced estrogen levels. The prevalence of hyperprolactinemia in psychiatric patients is between 30 and 70% (Alosaimi et al. 2018), significantly higher than the general population (Alosaimi et al. 2018). Age does not appear to influence prevalence rates of hyperprolactinemia in men, but a decline of prolactin level with age was observed in women (Montgomery et al. 2004), suggesting a postmenopausal effect. Although new data shows no age-related differences in prevalence (Alosaimi et al. 2018), a possible explanation for the inconsistencies may be explained by methodological differences, especially in the cut-off level for defining hyperprolactinemia.

In a comprehensive meta-analysis comparing 32 oral antipsychotics for the acute treatment of adults with multi-episode schizophrenia, it was discovered that olanzapine, asenapine, lurasidone, sertindole, haloperidol, amisulpride, risperidone, and paliperidone were associated with a significant elevation in prolactin levels (Huhn et al. 2019). A recent meta-analysis further demonstrated that both newer antipsychotics (such as risperidone, amisulpride, and paliperidone) and older antipsychotics (including chlorpromazine, haloperidol, and sulpiride) were linked to an increase in prolactin levels, showing a substantial effect size (Zhu et al. 2021). However, some newer antipsychotics, such as clozapine and aripiprazole, have demonstrated either no effect or a potential decrease in serum prolactin levels (Zhu et al. 2021; Kroigaard et al. 2022).

The impact of hyperprolactinemia, notably in its suppression of estrogen, has various physiological implications. In premenopausal women, antipsychotic-induced hyperprolactinemia often leads to menstrual disturbances, ranging from luteal phase insufficiency to irregular or infrequency menses, and sometimes amenorrhoea (Gleeson et al. 2016; Lee and Kim 2006; Seppala and Hirvonen 1976). Galactorrhea, either alone or in conjunction with menstrual disruptions, can also manifest (Melmed and Kleinberg 2003). Hyperprolactinemia may also explain the decreased fertility observed in men and women with schizophrenia (Edinoff et al. 2021). In women, prolactin inhibits follicle-stimulating hormone by suppressing gonadotropin-releasing hormone, impeding ovulation (Matsuzaki et al. 1994; Koike et al. 1991). Similarly, in men, prolactin inhibits gonadotropin release, affecting spermatogenesis (Dabbous and Atkin 2018). However, these are generally reversible upon restoration of normal prolactin levels. Additionally, reduced estrogen levels due to hyperprolactinemia have been shown to accelerate bone loss, heighten osteoporosis risk, and weaken bones, making fractures more likely (Mills et al. 2021; Papola et al. 2018). Hyperprolactinemia is also observed in antipsychotic-naïve patients. It is theorized that hyperprolactinemia in these patients could be explained by the activation of the stress response system. Stress has the potential to trigger the release of prolactin (Riecher-Rossler 2017), which subsequently prompts the secretion of dopamine, and this in turn can trigger psychotic symptoms. Recent research aligns with this hypothesis, demonstrating a correlation between elevated prolactin levels and more severe psychotic (Tharoor et al. 2020) and negative symptoms (Sayed El Taweel and Abdalla 2017) in antipsychotic-naïve patients. The role of stress in inducing hyperprolactinemia may have a stronger effect on women than men in emerging psychosis (Ittig et al. 2017; Hoekstra et al. 2021). This may be explained by estradiol having a stimulatory effect on prolactin response to stress (Caligaris and Taleisnik 1983; Poletini et al. 2006), while progesterone has an inhibitory effect (Caligaris and Taleisnik 1983; Jahn and Deis 1986). However, higher prolactin levels have also been reported in antipsychotic-naïve male than in women (Gonzalez-Blanco et al. 2016).

Regardless of whether hyperprolactinemia is caused by antipsychotic medication use or psychosocial stress, it should be screened in patients with schizophrenia. This is especially important for women with higher estrogen levels that should be maintained (i.e., premenopausal women), as women are generally more vulnerable to hyperprolactinemia (Gonzalez-Blanco et al. 2016). In this case, antipsychotic medications that either maintain or decrease prolactin should be favored over antipsychotics that increase prolactin (Gonzalez-Blanco et al. 2016).

## Estrogen as a treatment in schizophrenia

Individuals with schizophrenia, regardless of sex, often reach a point where their responses to antipsychotic medications stabilize. Unfortunately, there exists a notable portion of individuals who exhibit limited or no positive response to antipsychotic (Kane 2012). For such patients, considering innovative supplementary treatments like estrogen augmentation may hold significant importance.

### Human clinical trials in schizophrenia with estrogens

The efficacy of exogenous estradiol treatment as a clinical treatment in women with schizophrenia has been extensively demonstrated by our group (Kulkarni et al. 2008a; Kulkarni et al. 1996; Kulkarni et al. 2016; Kulkarni et al. 2015; Kulkarni et al. 2002). In 1996, we conducted the first trial of estradiol treatment for women with schizophrenia. We showed that the group receiving 20 mcg of adjunctive estrogen exhibited better recovery from acute psychotic symptoms than those who only received antipsychotics (Kulkarni et al. 1996). We then conducted subsequent double-blind, placebo-controlled study involving the adjunctive use of transdermal estradiol patches at dosages of 50 mcg, 100 mcg, and 200 mcg in women with schizophrenia (Kulkarni et al. 2015; Kulkarni et al. 2001; Kulkarni et al. 2008b). Our research consistently demonstrated that adjunctive estradiol treatment led to an improvement in psychosis symptoms in women, particularly with the use of 100 mcg transdermal estradiol. We also conducted a small-scale study involving men with schizophrenia, where adjunctive estradiol was found to have a positive impact on psychotic symptoms in this population (Kulkarni et al. 1999). Our findings have since been replicated in other clinical trials (Li et al. 2023; Akhondzadeh et al. 2003; Begemann et al. 2012). More recently, Weiser et al. (2019) found that transdermal estradiol is an effective adjunct treatment for women of childbearing age with schizophrenia. It also found improvements in negative symptoms and that the effect could be specific to those older than 38 years (Weiser et al. 2019).

Estradiol has also been found to be effective in improving cognition in women with schizophrenia compared with placebo (Ko et al. 2006). Cognitive improvements would be in line with studies suggesting a positive effect of 17-beta-estradiol on cognitive function in healthy ageing women

More recently, we highlighted for the first time that responses to estrogen treatment are heterogenous. In a repeated-measures study, we characterized the association between hormone levels (estrogen, progesterone, testosterone, prolactin, FSG, LH, DHEA) and symptom treatment outcomes (using the Positive and Negative Syndrome Scale (PANSS)) in women with schizophrenia taking adjunctive estradiol treatment (Thomas et al. 2021). From 56 patients, the results generated two subgroups: a treatment-responder group who demonstrated decreasing PANSS scores across time and a treatment-non-responder group, demonstrating stable PANSS scores across time. The data indicates that conducting multiple assessments of estradiol levels could pave the way for creating a molecular blood test. This test would be valuable in aiding clinicians in ascertaining whether endocrine modulation is a viable treatment approach for individual patients.

### Selective estrogen receptor modulators (SERMs)

The extended use of estrogen as an adjunctive treatment for women with schizophrenia raises potential concerns, particularly regarding its impact on breast and uterine tissue, as well as its feminizing effects in men (Kulkarni et al. 2012). Recent advancements in selective estrogen receptor modulators (SERMs) offer a promising avenue for intervention. SERMs offer the benefit of exerting antagonistic effects in breast and uterine tissues, while displaying agonistic effects in other tissues such as bone, and were primarily designed for the treatment of breast cancer and osteoporosis (Komm et al. 2005; Komm and Mirkin 2014).

Raloxifene, a form of SERMs, has been shown to be a safe and effective option for treating schizophrenia symptoms. Several double-blind clinical trials have demonstrated the effectiveness of raloxifene in improving symptoms of schizophrenia, including positive symptoms (Usall et al. 2011; Kianimehr et al. 2014), negative symptoms (Usall et al. 2011; Usall et al. 2016), general psychopathology (Kulkarni et al. 2016; Usall et al. 2011; Kulkarni et al. 2010), and cognitive functions (Weickert et al. 2015; Huerta-Ramos et al. 2014). A meta-analysis showed that raloxifene had significant positive effects on total symptom severity, as well as on positive, negative, and general PANSS subscales (De Boer et al. 2018). These findings are consistent with meta-analyses on raloxifene use in postmenopausal women with schizophrenia (Wang et al. 2018; Zhu et al. 2018). More recently, however, results from a double-blind, randomized clinical trial conducted by Brand et al. (2023) do not support the use of raloxifene in patients with schizophrenia-spectrum disorder in general. Instead, it was proposed that the effects of raloxifene may be dependent on sex, with a beneficial effect of raloxifene on negative symptoms only seen in women (Brand et al. 2023). These new findings warrant further exploration of the potential sex-specific effects of raloxifene.

Raloxifene has also been shown to enhance cognition. In a 12-week double-blind study, Huerta-Ramos et al. found significant differences in memory and executive function with the addition of 60 mg raloxifene (Huerta-Ramos et al. 2014). However, these effects were not replicated in a separate 24-week study (Huerta-Ramos et al. 2020). Research investigating the cognitive effects of high-dose raloxifene (120 mg) in healthy, postmenopausal women demonstrated a slight enhancement in verbal memory performance after 1 month of treatment (Nickelsen et al. 1999). These findings are consistent with those of Yaffe et al. (2005), who compared two doses of raloxifene (60 mg/day and 120 mg/day) with placebo in postmenopausal women exhibiting varying cognitive states. Their study revealed that women receiving a daily dose of 120 mg of raloxifene exhibited superior cognitive outcomes compared to those on 60 mg or the placebo (Yaffe et al. 2005).

These results are in line with our dose-finding study, which amalgamated data from both current and previous randomized controlled trials involving 35 postmenopausal women (Kulkarni et al. 2010). This analysis highlighted those participants receiving 120 mg, as opposed to 60 mg or placebo, experienced a significantly greater alleviation of psychotic symptom. Similarly, Weickert et al. found that a daily dose of 120 mg raloxifene improved cognitive function in young and middle-aged women as well as men with schizophrenia symptoms (Weickert et al. 2015), suggesting its positive action on cognitive extends to both sexes and across different age groups.

A recent narrative review failed to report raloxifene to have substantial effects on mood and cognitive symptoms in healthy postmenopausal women (González-Rodríguez et al. 2022). Instead, raloxifene may have a beneficial effect on sleep disorders, with women receiving raloxifene reporting better sleep quality compared to the control group (González-Rodríguez et al. 2022). Considering the common occurrence of insomnia in individuals with schizophrenia and the likelihood of higher rates of insomnia and other sleep disturbances in postmenopausal women with schizophrenia, it is possible that raloxifene may have positive effects in alleviating insomnia in this demographic.

Next-generation SERM — bazedoxifene — may have a greater impact on the CNS and therefore provide the consistent outcomes sought for people with schizophrenia. Bazedoxifene binds to both α and β intracellular estrogen receptor subtypes. Unlike raloxifene, it was developed with an indole-based structure featuring a 2-phenyl ring system as the core-binding unit, in contrast to the benzothiophene core (Kulkarni et al. 2019). This structural deviation accounts for bazedoxifene’s enhanced tissue selectivity when compared to other SERMs (Vestergaard and Thomsen 2010). A recent systematic review and meta-analysis affirm the substantial benefits of bazedoxifene in addressing bone-related concerns and osteoporosis, all without an increased risk of adverse or serious events, including myocardial infarction, stroke, venous thromboembolic event, or breast carcinoma (Peng et al. 2017). Moreover, it has been suggested that bazedoxifene has a safer breast health profile compared to other SERMs (Pickar and Komm 2015), which is extremely important for long-term use particularly in women.

Bazedoxifene has also been shown to cross the blood-brain barrier and enhance cognition. In mice, Hill et al. (2020) demonstrated that bazedoxifene can effectively enter the brain, activate neural estrogen response element in the brain, and acutely rescue ovariectomy-induced spatial memory deficits. The findings suggest bazedoxifene could be a viable cognitive enhancer with promising clinical applicability. Kulkarni and colleagues are currently conducting the first clinical trial examining the impact of bazedoxifene as an adjunctive treatment in the treatment of women with schizophrenia.

### Hormonal contraceptives and schizophrenia

Most women with schizophrenia are of reproductive age. Hormonal contraceptives, including the oral contraceptive pill, are an effective and easy contraceptive method that has provided women with a greater degree of control over their reproductive lives for centuries. However, very few women with schizophrenia seek contraceptive advice, lacking sufficient information about contraception options. Many women with schizophrenia are sexually active and research indicates a prevalence of unwanted pregnancies ranging between 24.3 and 47.5% within this population (Posada Correa et al. 2020). Considering the challenges of daily pill adherence among women with schizophrenia, alternative contraceptive methods such as intrauterine devices, depot injections of progesterone, and tubal ligation are often recommended (Miller and Finnerty 1998).

Despite the extensive body of research examining estrogen’s impact on schizophrenia and cognition, as well as the broader effects of oral contraceptives on cognition, there is limited research investigating the specific influence of oral contraceptives in schizophrenia. However, the combined oral contraceptive pill is clinically employed as an estrogen treatment for younger women diagnosed with schizophrenia. Addressing this gap requires randomized controlled trials specifically focusing on oral contraceptive use in women with schizophrenia.

Importantly, it has been recently shown that in women who are susceptible to mood disorders, including schizophrenia, hormonal contraceptives may precipitate or perpetuate depression (Mu and Kulkarni 2022). In particular, older oral contraceptive pills containing ethinylestradiol, the estrogen component of the pill, are linked to severe mood problems, whereas newer versions containing physiological forms of estrogen may be better tolerated and show a weaker association with mood problems. This highlights the importance for clinicians to carefully evaluate the contraceptive formulation and timing concerning the onset or exacerbation of mood changes in susceptible individuals, including those with schizophrenia.

## Clinical recommendations and future directions

The key clinical recommendations are to ensure that a menstrual history is taken in all women who present with psychosis to determine whether there is an association between their menstrual cycle and the severity of psychotic symptoms.

It is also important to recognize the likelihood of relapse or new onset of psychotic illness during perimenopause, especially among women with a history of exacerbated symptoms during the menstrual cycle or postpartum period. This is especially crucial as these women appear particularly vulnerable to mental state fluctuations linked to hormonal shifts. The potential therapeutic benefits of menopause hormone therapy can be explored in these women who may significantly benefit from estrogen augmentation. This approach is preferred compared to increasing the dosage of antipsychotic medications to counteract the exacerbated symptoms and reduced enzyme production responsible for metabolizing these medications caused by decreased estrogen levels. Relatedly, it is crucial to develop safe and effective approaches for reducing antipsychotic doses to minimize risks associated with hyperprolactinemia, decreased bone density, heightened chances of heart disease and stroke, and increased cancer mortality rates. Restoring adequate estrogen levels is essential to ameliorate these consequences.

In relation to future research, there is a need for more studies and randomized controlled trials in women with schizophrenia. Understanding the efficacy and impact of estrogen treatments, such as oral contraceptive pills and newer generation SERMs like bazedoxifene, in women with schizophrenia is crucial for clinicians and researchers alike. Conducting randomized controlled trials focused on these treatments will offer valuable insights into their effectiveness, safety, and potential as adjunctive therapies for managing psychotic symptoms.

From a mechanistic point of view, future research probing the links between decreased estrogen and psychotic symptoms is crucial to better understand how hormonal changes during the menopausal transition affect the brain and psychotic symptoms, as well as understanding variations between individuals in their sensitivity to hormonal changes. In addition to the direct impact of hormonal changes during menopause, it is important for future research to explore the additional effects of other menopausal symptoms on overall well-being and the role of other menopause symptoms in psychosis. For example, while the effects of lower estrogen and psychotic symptoms are well documented, the relationship between vasomotor symptoms and psychosis in menopause has not been explored. Expanding this understanding will assist clinicians to anticipate, recognize, and address potential mental health challenges during menopause, contributing to more tailored and effective treatment strategies for women experiencing both vasomotor and psychotic symptoms.

## Conclusion

The promising findings regarding hormone treatments in psychosis present a compelling avenue for future research and clinical interventions. Leveraging estradiol as a “window to the brain” holds great potential for deepening our understanding of the underlying causes of psychotic disorders. For individuals with schizophrenia, especially those who have limited response to antipsychotics, adjunctive estrogen could be considered. The research suggests that this approach may lead to improved symptom management and cognitive function. Additionally, it is imperative to acknowledge the unique impact of menopause on women with schizophrenia, highlighting the critical need for targeted hormone treatments to proactively prevent relapses. Emphasizing women’s mental health as a distinct neurobiological domain is paramount, as it provides a refined framework to inform more effective and tailored treatments. This approach not only advances the field of psychiatry but also holds the potential of significantly improving the well-being and outcomes of women affected by psychosis. The risk and benefits of short- and long-term use of hormone therapy should be discussed prior to commencement of any form of estrogen therapy.
